# Notes on New Remedies

**Published:** 1871

**Authors:** John Stainback Wilson

**Affiliations:** Atlanta, Georgia


*Notes on New Remedies.* By JOHN STAINBACK WILSON, M.D., of Atlanta, Georgia.

So numerous are the innovations of modern pharmacy and therapeutics that it requires no inconsiderable diligence for the student of medicine to acquire and retain even the names of the new remedies, and the new combinations and active principles of old remedies which our industrious and money-loving druggists and pharmaceutists are daily urging on the attention of the medical profession. Not only do new remedies abound, but new modes of treatment are sufficiently prevalent and authoritative to unsettle many of the time-honored and apparently well established principles which have heretofore guided and governed us in the treatment of disease.

In proof of this it is only necessary to refer to the treatment of the second stage of pneumonia by opiates and stimulants, as advocated by Professor Flint and others, instead of by blisters and mercury to ptyalism, as taught by almost all our medical authors for many years past.

Another notable change in our theapeutics is the abandonment of blood-letting, not only in pneumonia, but in almost every other disease, and the admission that when it is practiced, it is more as a prompt palliative than as a positive curative agent. In other words, the doctrine is inculcated from high places, and I believe generally admitted, that venesection cannot abort or cut short inflammation when fully developed.

The doctrine that typhoid fever, the eruptive fevers, and several other diseases, cannot be cut short, is now admitted, and acted on by all well informed physicians. Hence spoliative, depressing and dangerous remedies are abandoned, not only in the diseases above mentioned, but in almost all diseases, and a stimulating, sustaining and even a do-nothing expectant plan of treatment is very generally adopted.

Whatever may be said as to the fixedness of medical science, it can hardly be assumed that therapeutics is a fixed science. For, in these modern days, we are all afloat as to the treatment of disease, drifting along on an apparently boundless sea of transition, the waves of change following each other in such rapid succession as to make us dizzy.

But we have no reason to despair of the ship. For though all changes are not progress, and though many of the novelties of the day will doubtless sink into oblivion under the test of time and experiment, yet our course is upward and onward, and the most incredulous and iron-sided conservative must admit that our means for alleviating and even curing disease, have been greatly increased by new modes of treatment, and by the addition of new remedies made to our pharmacopia within the past few years.

Among the new modes of treatment, nothing is more indicative of progress in the right direction than the substitution of a sustaining for a depletive and reducing plan of treatment ; reliance on the power of nature, and the giving of more attention to diet and other hygenic agents, to the exclusion of excessive drugging.

We are at last beginning to learn the great lesson that nature cures disease, and that we can do no more with our drugs and other appliances than to assist and sustain her in the contest. Who will say that this is not a great advance in knowledge? And who can estimate the deaths that have resulted from opposite views?

But while the different modes or principles of treating disease is a fruitful theme, the various remedies which have been added to our Materia Medica, open up a still larger field. Indeed, to discuss these in detail would require a volume.

But in these hurried notes, I will attempt nothing more than a brief sketch of some of the more prominent modern theapeutic agents. All of these are not strictly new, but some of them being practically unknown through disuse and consequent ignorance of their remedial virtues, may be properly regarded as new remedies.

Under this head comes Veratrum Viride, one of the most precious resources of modern therapeutics. Until Dr. Norwood discovered the potent sedative properties of this remedy, some twenty odd years ago, it, like hundreds of other things, was regarded as a useless cumberer of our overburdened Materia Medica.

Soon after the claims of this medicine were brought before the profession by Dr. Norwood, I prepared a tincture according to his formula, and used it in a variety of diseases with the most satisfactory results, which were reported to the *Southern Medical and Surgical Journal*, Augusta, Ga.

I administered it during the exacerbation of periodical fevers, in typhoid and puerperal fevers, in the different phlegmasiæ, especially in pneumonia and bronchitis, and in one case of chorea attended with inordinate excitement of the circulation. Indeed, I did not hesitate to use it in most cases of excessive cardiac action, to control the circulation, while other remedies indicated by the nature of the disease were not neglected.

Some may object to this practice as useless or dangerous. But surely a remedy will not be considered useless which will moderate excessive action of the heart, relieve acute hyperemia when present, and guard against the supervention of congestion or inflammation so likely to occur in the progress of fevers and other diseases; and all this without the loss of blood, with its long continued depressing effect.

As to the safety of the Veratrum, extended observation of its action authorizes me to affirm that the dose can be so nicely regulated, the effects are so uniform, manifest and palpable as to enable us to give our directions with a definiteness, mathematical precision and consequent safety that can scarcely be claimed for any other remedy.

Veratrum is one of the few drugs in the administration of which we can have the unerring guide of figures as to the regulation of the dose. And so uniform and certain is its sedative action, that we can confidently predicate our directions on this result.

Therefore, all we have to do in the administration of the remedy is to begin with a minimum dose and gradually increase until the desired reduction of the pulse is accomplished, as counted by the watch. My practice has been to begin with two or three drops for an adult, repeated every three hours, increasing one drop each dose until the pulse is reduced to the normal standard. When this is attained, I diminish the dose to one drop, and discontinue, if the pulse sinks below the point mentioned. By persuing this course, I have succeeded in effecting the desired sedation in almost all cases, without nausea, vomiting or other disagreeable symptoms.

Close observation has taught me that three hours, as a general rule, is the proper interval between doses, the previous dose requiring support in about this time.

Though my object is to give a general or bird's-eye view of the results obtained by the introduction of certain new remedies, rather than to enter into specific details, yet I have deemed it advisable to say this much on the manner of administering one of the most important of these remedies, because I have good reason to believe that the effects which have given rise to fears as to its safety, have originated from prescribing it in too large or too frequently repeated doses.

Like all other potent remedies, Veratrum should be used with judgment and discrimination.

Guided by these in the selection of proper cases for its administration, and pursuing the course above indicated as to dose, the physician can use this great sedative with a certainty, safety, success and satisfaction which he can hardly hope to realize from any other remedy.

Assuming, then, that this position will be sustained by the concurrent testimony of the numerous observers who have tested the remedy for several years past, I pass to the consideration of some other modern additions to our pharmacopea.

It is equally unnecessary to enlarge on the incalculable benefit conferred on suffering humanity by the introduction of the anæsthetics, and especially of the great anti-spermodic and pain-annihilator—chloroform.

Future experiments may demonstrate the superior anæsthetic virtues of bichloride of methylene, or some other article of its class; yet will blessings ever be breathed with the name of the recently departed discoverer of chloroform, the greatest boon of modern science to mankind.

Our Medical Journals teem with cases of various kinds of disease relieved by this remedy, and doubtless further investigations will extend its range of usefulness, especially when administered in the fluid form or *per orem.* And in this connection I would call attention to its recommendation by Dr. A. P. Merrill, of Memphis, as an abortive in chills, and more particularly in what are called congestive chills. Dr. Merrill gives it in these cases in teaspoonful doses, and speaks in high terms of its efficacy.

I may also add that in a recent discussion in the Atlanta Academy of Medicine, Dr. Miller stated that he had been in the habit of using it in puerperal convulsions, not only as an anti-spasmodic, but as a preventive or corrective of the albumenuria, so often associated with this affection.

Should future observations demonstrate this power, another great advance will have been made in modern theapeutics.

The bromide of potassium is another remedy whose virtues have not been properly appreciated until a recent date.

The last edition of the United States Dispensatory says: "It is thought to be sedative to the nervous system, and powerfully anti-aphrodisiac."

Our medical periodicals abound with the most conclusive evidence that the surmise is true, and that the bromide has wonderful power in controlling the gravest forms of convulsive disease.

Did time and space permit, I might speak of chloral, carbolic acid, viburnum, fluid extracts, active principles, inhalations, hypodermic medication, and many other additions to modern therapeutics, and improved modes of treatment, but this must suffice for the present.

At some future time this interesting and inexhaustible subject may be resumed, under circumstances more favorable to its discussion than those which have attended these hasty notes.

